# Influence of Hypoxia on the Epithelial-Pathogen Interactions in the Lung: Implications for Respiratory Disease

**DOI:** 10.3389/fimmu.2021.653969

**Published:** 2021-03-24

**Authors:** Lee K. Page, Karl J. Staples, C. Mirella Spalluto, Alastair Watson, Tom M. A. Wilkinson

**Affiliations:** ^1^ Clinical and Experimental Sciences, University of Southampton Faculty of Medicine, Southampton, United Kingdom; ^2^ NIHR Southampton Biomedical Research Centre, Southampton Centre for Biomedical Research, Southampton General Hospital, Southampton, United Kingdom; ^3^ Birmingham Medical School, University of Birmingham, Birmingham, United Kingdom

**Keywords:** epithelial cells, hypoxia, hypoxia-inducible factor (HIF)-1, host-pathogen interactions, innate immunity, respiratory disease

## Abstract

Under normal physiological conditions, the lung remains an oxygen rich environment. However, prominent regions of hypoxia are a common feature of infected and inflamed tissues and many chronic inflammatory respiratory diseases are associated with mucosal and systemic hypoxia. The airway epithelium represents a key interface with the external environment and is the first line of defense against potentially harmful agents including respiratory pathogens. The protective arsenal of the airway epithelium is provided in the form of physical barriers, and the production of an array of antimicrobial host defense molecules, proinflammatory cytokines and chemokines, in response to activation by receptors. Dysregulation of the airway epithelial innate immune response is associated with a compromised immunity and chronic inflammation of the lung. An increasing body of evidence indicates a distinct role for hypoxia in the dysfunction of the airway epithelium and in the responses of both innate immunity and of respiratory pathogens. Here we review the current evidence around the role of tissue hypoxia in modulating the host-pathogen interaction at the airway epithelium. Furthermore, we highlight the work needed to delineate the role of tissue hypoxia in the pathophysiology of chronic inflammatory lung diseases such as asthma, cystic fibrosis, and chronic obstructive pulmonary disease in addition to novel respiratory diseases such as COVID-19. Elucidating the molecular mechanisms underlying the epithelial-pathogen interactions in the setting of hypoxia will enable better understanding of persistent infections and complex disease processes in chronic inflammatory lung diseases and may aid the identification of novel therapeutic targets and strategies.

## Introduction

The airway epithelium is located at the interface between the internal and external environment and is strategically positioned to interact with the environment in a dynamic fashion. The pseudostratified mucosal barrier consisting of multiple cell types, constitutes the lung epithelium (1). In a healthy state the airway epithelium plays an integral role in host defense through a physical and mechanical barrier, innate immune mediator production, and chemokine and cytokine production to recruit inflammatory cells for both propagation and resolution of the immune response (2–4). The importance of the lung epithelium is exemplified in chronic inflammatory lung diseases, where epithelial cell dysfunction is associated with compromised immunity and chronic inflammation in the lung (5–9). Structural and functional abnormalities in both the airway and alveolar epithelium have a significant impact on host defenses, immune/inflammatory response, and the repair process leading to progressive lung damage and impaired lung function.

Although an oxygen rich environment under normal physiological conditions, the lung mucosal surface is susceptible to conditions of oxygen deficiency or tissue hypoxia (10) during infection and inflammation, which occurs when cellular demand exceeds supply. Direct *in vivo* evidence has demonstrated that pulmonary infection is associated with profound local hypoxia (11–14). The occurrence of hypoxia during infection and associated inflammation is multifaceted and involves increased oxygen demand in order to satisfy the requirements of inflamed resident cells, and in some instances, multiplying pathogens (15–17). Furthermore, infiltrating inflammatory cells such as neutrophils are thought to influence the tissue environment due to their metabolic cost. For example, it has been demonstrated that migration of neutrophils across the epithelium increases the transcriptional activity of hypoxia-inducible genes in epithelial cells, due to localized oxygen depletion, resulting in microenvironmental hypoxia which in turn, influences the resolution of inflammation (18). Furthermore, beyond acute infection, chronic inflammatory respiratory diseases are also commonly associated with mucosal hypoxia. The airways of respiratory disease patients are characterized by chronic inflammation, structural changes and fibrosis, and airways obstruction through excessive mucus accumulation (19–22), which can lead to regions of local tissue hypoxia. Cystic fibrosis (CF) is an autosomal recessive disorder caused by mutations in the CF transmembrane conductance regulator (CFTR) gene. CF is characterized by airway mucus plugging, reduced mucus clearance due CFTR defects which renders the CF airways vulnerable to chronic infection and inflammation. The mucus filled CF airway, infected with *P. aeruginosa* is extremely hypoxic (23). It is thought that thick stagnant mucus infiltrated with immune cells and multiplying pathogens creates a steep oxygen gradient within the mucus, exposing the underlying epithelial cells to marked hypoxia. Furthermore, the airway epithelium of mouse models of CF stained strongly with the specific hypoxia probe, pimonidazole hydrochloride (Hypoxyprobe, which binds at a threshold of ≤ 10 mmHg O_2_) (24), confirming that tissue hypoxia is present in this inflamed airway epithelium. Chronic obstructive pulmonary disease (COPD) is characterized by chronic airway inflammation and functional and structural alterations in the lung, primarily caused by long-term inhalation of harmful particles such as cigarette smoke (20, 25, 26). Remodeling in the large airway in COPD, is accompanied by thickening and fibrosis of the subepithelial microvasculature and perivascular fibrosis (27), which may significantly reduce oxygenation of the airway epithelium. Increased expression of hypoxia-inducible factor (HIF)-1α is detected in the bronchial epithelium in COPD in areas of airway remodeling and goblet cell hyperplasia (28–30). Asthma, is another obstructive airway disease that involves chronic airway inflammation of the respiratory tract and excessive mucus production which is triggered by a variety of airborne insults including allergens, dust, smoking and respiratory pathogens. The increased expression of HIF-1α in lung mucosal biopsy specimens from asthmatic patients (31), may also indicate the presence of a tissue hypoxia in the asthmatic airway.

Pulmonary diseases associated with infection, excessive airway inflammation, airway obstruction, airway remodeling and emphysema can lead to decreased blood and also tissue oxygenation and consequently a fall in the partial pressure of oxygen in the arterial blood (10, 32, 33). This is particularly evident in COVID-19 where hypoxia is a major risk factor for pneumonia and respiratory distress following severe acute respiratory syndrome coronavirus 2 (SARS-CoV-2) infection (34, 35). Furthermore, in COPD, the progression of the disease increases the risk of alveolar hypoxia and consequent hypoxemia (36). Ventilation/perfusion (V/Q) mismatch resulting from progressive airflow limitation and emphysematous destruction of the pulmonary capillary bed is the main factor contributing to hypoxemia in COPD patients (36). Hypoxemia associated with COPD contributes to reduced quality of life, diminished exercise tolerance, reduced skeletal muscle function, and ultimately increased risk of death (37). Moreover, exacerbations of COPD, which are associated with disease morbidity and mortality (38–41), are also frequently associated with deterioration in gas exchange and associated hypoxemia, due to increased tissue oxygen consumption and V/Q mismatch (42).

Whilst local tissue hypoxia and systemic hypoxia in the lung play a prominent role during infection and is present in chronic inflammatory respiratory diseases, the role of hypoxia at the level of the tissue in shaping the host-pathogen interactions in respiratory diseases is not fully understood. Here we review the current evidence around the role of tissue hypoxia in modulating the host-pathogen interaction at the lung epithelium. Furthermore, we highlight the essential work now needed to outline the role of tissue hypoxia in the pathophysiology of inflammatory lung diseases and emerging lung diseases such as COVID-19 and post-COVID-19 chronic lung disease.

## Transcriptional Responses to Hypoxia

**Figure 1:**
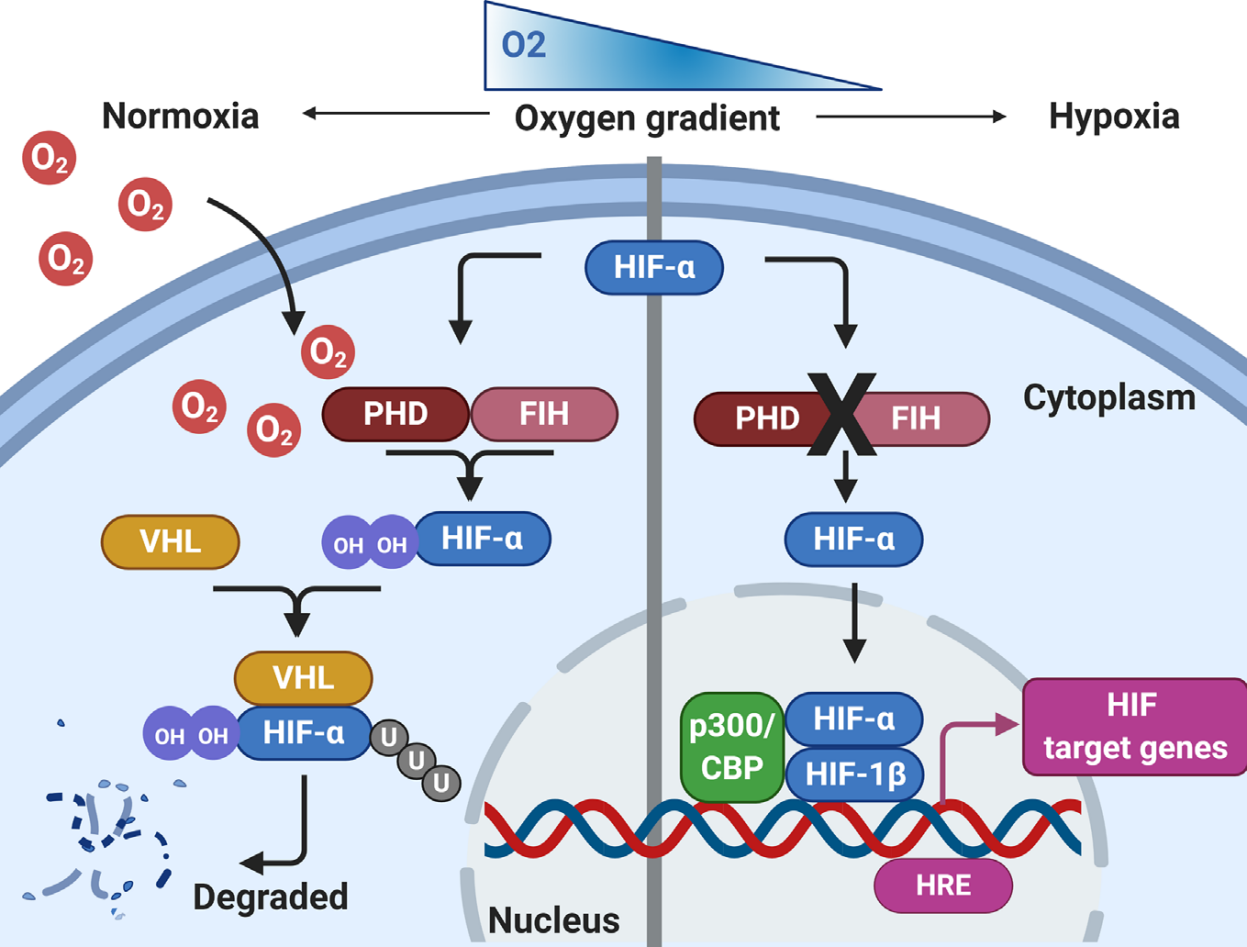
Regulation of HIF by hypoxia. During normoxia, HIF-α is hydroxylated by prolyl hydroxylase domain proteins (PHD) and factor inhibiting HIF (FIH), using molecular oxygen. This leads to HIF-α interacting with Von Hipple-Lindau (VHL), before being targeted for proteasomal degradation. During conditions of hypoxia, HIF-α hydroxylation by PHD/FIH is inhibited and HIF-α is not targeted for proteasomal degradation. HIF-α can translocate to the nucleus, where it binds with HIF-1β and recruits co-activators at the hypoxia response element (HRE) to initiate gene transcription. Created with BioRender.com.

For the host to be able respond to pathogens effectively and efficiently in hypoxic conditions, certain hypoxic regulatory mechanisms are essential. Thus, it is important to fully appreciate the significant effect that hypoxia has on downstream signaling pathways in tissue resident and infiltrating immune cells. The immune response under hypoxic conditions is governed by several pathways and metabolic activity, of which hypoxia-inducible factor (HIF), is the best characterized and termed the master regulator of the host response to hypoxia (**Figure 1**). HIF is a DNA-binding transcription factor that associates with specific nuclear cofactors during hypoxia. HIF regulates hundreds of downstream genes which are involved in diverse biological pathways (43). The regulatory complex is comprised of HIF-1β, a constitutive subunit, and one of the HIF-α isoforms: HIF-1α or HIF-2α (44). In the presence of oxygen, HIF-α subunits undergo hydroxylation by prolyl hydroxylase domain (PHD) proteins and an asparagine hydroxylase known as the factor inhibiting HIF (FIH), in an oxygen and iron-dependent manner (45). Hydroxylated HIF-α is then targeted by the von Hippel-Lindau (VHL) protein, a substrate-recognition subunit of an ubiquitin-protein ligase which subsequently interacts with HIF-α to undergo proteasomal degradation (46). During hypoxia, the activity of hydroxylase is reduced, leading to HIF stabilization, translocating to the nucleus from the cytoplasm, ultimately dimerizing with the HIF-1β subunit, to form the active HIF complex (47). Finally, the active forms of HIF recruit coactivator proteins to the hypoxia-response element, activating the transcription of target genes required for undergoing adaptations to hypoxia. HIF-1α is widely expressed in many innate immune cells including macrophages, neutrophils, dendritic cells and epithelial cells. Integral innate immune functions are preserved in hypoxic conditions through HIF. For example, the survival of neutrophils is extended in hypoxic conditions through HIF-1α and nuclear factor kappa-light- chain-enhancer of activated B cells (NF-κB) activation (48). Furthermore, it has been demonstrated that hypoxia-mediated HIF-1 activation of NF-κB in airway epithelial cells releases greater amounts of proinflammatory cytokines (49). However, hypoxia may also suppress the epithelial inflammatory response (50, 51). Exposure of murine airway epithelial cells to hypoxia has been shown to reduce the expression of key innate immune molecules through reduced NF-κB signaling (51). Moreover, the macrophage response to bacterial lipopolysaccharide (LPS) is enhanced under hypoxia, through HIF-1-mediated, increased toll-like receptor (TLR) gene expression, resulting in increased expression of cyclooxygenase-2, interleukin-6 and Regulated on Activation, Normal T Cell Expressed and Secreted (RANTES) (52, 53), suggesting that hypoxic conditions may contribute to the aggravated inflammatory responses during infection. Hypoxia also regulates other key transcription factors which contribute to the overall cellular response, including the major regulator of immunity NF-κB (54). Hypoxia-mediated activation of NF-κB is through decreased PHD-dependent hydroxylation of inhibitor of nuclear factor kappa B kinase subunit beta (IKKβ), resulting in the phosphorylation-dependent degradation of IκBα and liberation of NF-κB. Therefore, HIF-dependent and HIF-independent regulatory pathways in host cells that are regulated during tissue hypoxia may impact on the progression of chronic inflammatory respiratory diseases. Although hypoxia is essential in driving immunological processes and resolving respiratory infections, the relationship between immunity and hypoxia is delicately balanced. Resultant sustained aberrant inflammation and activity of immune cells leads to tissue damage and is a pathological hallmark of many chronic inflammatory respiratory diseases.

## Hypoxia-Epithelial Interactions: Consequences for the Host During Respiratory Infection

**Figure 2:**
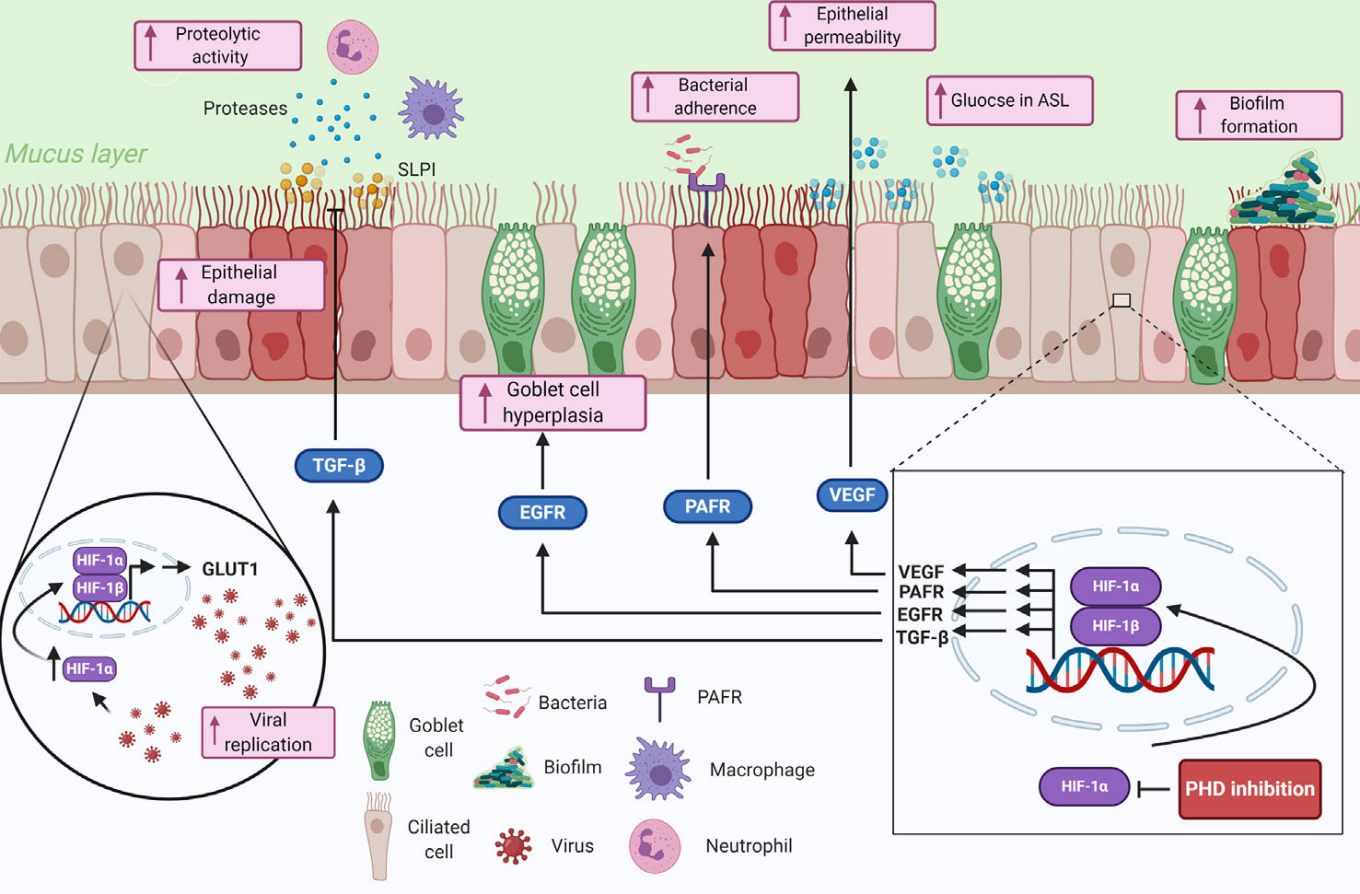
Summary of the potential contributions of tissue hypoxia to the epithelial-pathogen interactions in respiratory diseases. Hypoxia is sensed by airway epithelial cells, that proceed to upregulate genes involved in the response to hypoxia through HIF-1α. The upregulated genes and proteins in response to HIF-1 transcription modulate several immune responses including impairing epithelial barrier function, reducing mucociliary clearance, modulating nutrient availability, and reducing anti-proteolytic enzymes. Hypoxia could also play important roles in pathogen colonization of the lung epithelium by mediating bacterial adherence through HIF-1-dependent mechanisms and enhance biofilm formation through bacterial-adaptive mechanisms. Finally, viral infected epithelial cells can be manipulated to upregulate glycolytic pathways through a HIF-1α-dependent mechanism, which consequently increases viral replication in the cells. Created with BioRender.com.

The airway epithelium forms the interface between the external environment and the internal milieu, making it a prime target for inhaled pathogens. However, the epithelium is not just a bystander, it forms an integral part of the innate immune system through being a physical barrier as well as releasing effectors to initiate and orchestrate immune and inflammatory responses (1). Furthermore, dysfunction of the airway epithelium is associated with inflammatory lung diseases and increases the susceptibility to infection (55). Here we will discuss how hypoxia influences the epithelial-pathogen interactions in the lung, with important implications for respiratory disease (**Figure 2**).

### Mucus Hypersecretion and Reduced Mucociliary Clearance

A fundamental lung mucosal defense mechanism is the secretion of mucus into the bronchial airway lumen to capture and trap invading pathogens, before being mechanically removed *via* mucociliary clearance (56, 57). The mucosal layer in the conducting airways exhibits a continuous layer of secreted proteins called mucins. Mucins are large glycoproteins formed of O-linked polysaccharides, which form a fluid-like barrier tethered to epithelial cells (57). The mucus-producing goblet cells function as the primary secretory cell, with the more abundant ciliated cells functioning as transporters to move the mucus up the airways for removal (58, 59). The ratio of secretory to ciliated cells is regulated both during normal physiological processes and also during infection to maintain optimal mucociliary function (60). However, chronic respiratory diseases are associated with pathological changes with the development of goblet cell hyperplasia, reduced expression of ciliated cells, mucus hypersecretion and mucus plugging (61–65). Sustained activation of disease associated signals such as from cigarette smoke, allergens and pathogens, promote the excessive differentiation towards goblet cell hyperplasia and mucus hypersecretion (66). Consequently, increased mucus production, dehydration of the airway surface liquid and mucus plugging prevents the mucociliary functions of the innate immune system to adequately clear the airways of mucus and pathogens (67, 68).

The development of mucus plugs has also been associated with cellular hypoxia of epithelial cells lining the airways, creating hypoxic niches within the adherent mucus and subjacent epithelial cells (24). This is exemplified by mucus obstructed airway epithelial samples from COPD patients which are known to be hypoxic, exhibiting an increased expression of HIF-1α in areas of goblet cell hyperplasia, and the hypoxic mucus filled CF airways (23, 69). Furthermore, analysis of CF and COPD lung tissue samples found that hypoxic airway epithelial necrosis in the mucus obstructed airways was a key trigger for neutrophilic inflammation, which may further exacerbate epithelial hypoxia (70). Interestingly, HIF-1α has been shown to induce goblet cell hyperplasia and increase the expression of the mucin MUC5AC, *via* the epidermal growth factor receptor (EGFR) (71–73). The EGFR is upregulated in the diseased airway epithelium and aberrant EGFR signaling has been implicated in the pathogenesis of asthma, COPD and CF (74, 75). EGFR is an important protein involved in the epithelial repair process *via* the induction of epithelial migration, proliferation, differentiation, and extracellular matrix synthesis. However, the activation of EGFR, also leads to goblet cell hyperplasia, mucus hypersecretion, and overproduction of MUC5AC, which may exacerbate the pathological changes in respiratory diseases (76–79). Overproduction of the MUC5AC gene is a characteristic of many respiratory diseases including CF, asthma, and COPD (7, 80, 81), creating hyper-concentrated mucus, which is thicker, more adherent and prone to mucus plugging (82). Consequently, thicker and more adherent mucus may create further hypoxic niches within it. This has major implications not only for the progression of diseases but also for the host-pathogen interactions within the airways. Several bacterial pathogens, including non-typeable *Haemophilus influenzae* (NTHi), *P. aeruginosa and Staphylococcus aureus*, are able to bind to and utilize mucins as a mechanism for colonization of the lung (83–85). Additionally, the hypoxic niches created in the mucus plugged airway may favour infectious organisms that gain energy efficiently under anaerobic conditions (86, 87). The increased presence of anaerobic bacteria in the airway at more advanced stages of respiratory diseases have been demonstrated in lung microbiome studies (88–90). The changes in microbiota in the diseased airway may negatively impact the progression of the disease (91–93). For example, *P. aeruginosa* which is found in sputum more frequently in advanced stages of COPD is associated with exacerbations and increased risk of mortality (94, 95). Furthermore the presence of obligate anaerobes have been linked to disease severity and inflammation in CF (96, 97). Although still unknown, it may be postulated that mucolytic therapies which clear the airways of sputum, reduce mucus production and plugging and prevent airway obstruction could also play a role in reducing mucosal hypoxia. Alternatively, interventions which correct local hypoxia e.g. long-term oxygen therapy could hypothetically improve mucosal regulation and mucus hypersecretion and warrants investigation.

### Disruption of the Epithelial Barrier

An important function of epithelial cells is to act as a physical barrier towards the outside environment. Tight junctions (TJ), adherens junctions (AJ) and desmosomes form the transcellular junctions (98). These junctions are formed through intercellular junctional proteins including claudins, connexins, paranexins, cadherins, adhesions, and zonula occludins (ZO), which link to the actin cytoskeleton (99, 100). This tightly regulated physical barrier not only controls paracellular ionic movements and non-permeability of the epithelium (101, 102), but also prevents microbial compounds and airborne substances access to the body interior. The epithelial barrier is critical in the innate host defense as a loss of barrier function increases the susceptibility of the host to infection and injury by pathogens and proteases (103). In addition, a pathological hallmark of several chronic inflammatory respiratory diseases, including asthma and COPD, is impaired epithelial barrier function and increased epithelial permeability, which may permit access for pathogens to the underlying submucosa (5, 7, 104–107). A number of factors have been implicated in epithelial barrier dysfunction including infection (108–110) and inhalation of noxious particles such as cigarette smoke (111). Several studies have also demonstrated that exposing airway epithelial cells to hypoxia decreased the expression of apical cytoskeleton proteins actin and α-spectrin (112), and also the expression of the TJ proteins ZO-1, claudin-4, occludin, and E-Cadherin (49, 112, 113), which consequently reduced epithelial barrier function. One possible mechanism is through the HIF-1α-mediated induction of vascular endothelial growth factor (VEGF), during hypoxia, which has been shown to increase epithelial permeability (114). VEGF is a pleiotropic protein that regulates vascular angiogenesis and endothelial permeability and is an important adaptive mechanism to hypoxia, enhancing local vascularization and oxygen transport (115), in addition to being an important mediator of inflammation (116). The expression of VEGF is upregulated in the diseased airway epithelium, and has been implicated in airway remodeling processes (28, 117–119). Additionally, VEGF expression itself in the epithelium is a good indicator of local tissue hypoxia. Several studies have demonstrated that exposing airway epithelial cells to hypoxia, increased the expression of VEGF *via* HIF-1 (114, 120–122). Though the induction of VEGF may be an adaptive response of angiogenesis when oxygen availability is reduced; paradoxically this may have pathological consequences in terms of epithelial barrier disruption. Thus, the hypoxia-HIF-1α-VEGF axis may be an important mechanism driving epithelial barrier disruption and increased permeability of the epithelium. Regarding the lung mucosal defense mechanisms, vulnerability to adherence and invasion of pathogens is increased by the leaky epithelial barrier, resulting in airway infection and subsequent inflammation. Therefore, hypoxia-mediated epithelial permeability may ultimately facilitate pathogen invasion of the epithelium and persistence in the airway epithelium.

### Increased Bacterial Adherence to the Epithelium

A crucial step for effective bacterial colonization and invasion involves adherence to host components in the airways (123, 124). Platelet-activating factor receptor (PAFR) is one of the main epithelial cell-derived adhesion molecules used by both Gram-positive and Gram-negative bacteria (125–129). PAFR is a G-protein coupled epithelial cell membrane receptor that naturally binds the phosphorylcholine (ChoP) ligand on the eukaryotic proinflammatory chemokine PAF (130). Several species of airway bacteria display a host-derived zwitterionic/bipolar molecule ChoP on their bacterial walls, mimicking PAF to facilitate adherence to epithelial cells (131, 132). PAFR is upregulated in the lungs of asthmatic and COPD patients (133–135). The expression of PAFR is upregulated by noxious environmental particles such as cigarette smoke, electronic cigarettes, and biomass smoke exposure and exposure to pathogens (126, 128, 134, 136–139). Additionally, hypoxia prominently induces epithelial PAFR through HIF-dependent mechanisms (140). The upregulation of PAFR in the lungs has been associated with increased adherence to the mucosal surfaces by respiratory pathogens, airway inflammation, speed of lung function decline, and development of pneumonia (126, 136, 138, 141). Ultimately, microbial manipulation of PAFR, may be an important strategy for successful colonization and infection. The importance of ChoP-PAFR mediated bacterial adherence to epithelial cells has been confirmed by the use of PAFR antagonists, which have been shown to reduce bacterial adherence and invasion (127–129, 137). Thus, the dynamic and integral role of HIF-1α in key immune functions opens complex questions regarding HIF-1α. However, there is still much needed research regarding the role of HIF-1α and PAFR.

### Enhanced Biofilm Formation

The formation of multicellular microbial communities called biofilms is a critical step for pathogens during colonization of the lung, enabling survival and persistence in the challenging environment by attaching to a living surface (142, 143). Many different microbes reside in biofilms and the majority of persistent infections involve biofilms (144). Notably, biofilm communities enhance antimicrobial resistance to exogenous and endogenous effector molecules (145). There are clear associations between biofilm formation, inflammation, and respiratory disease and biofilms have been implicated in the pathogenesis of COPD and CF (143, 144, 146, 147) and developing bronchopneumonia (148, 149). Hypoxic conditions in the diseased lung may provide prime conditions for biofilm formation by *P. aeruginosa* which produce greater amounts of alginate under anaerobic conditions, a component involved in biofilm formation and protection against host immune responses (23, 150). Additionally, *P. aeruginosa* grown under anaerobic or hypoxic conditions yields greater antibiotic resistance and biofilm formation suggesting that hypoxia may be a crucial component in bacterial persistence (151, 152). The increase in antibiotic resistance appears to be due to hypoxia altering the stoichiometry of multidrug efflux pumps (153). An important mechanism of antibiotic resistance is the expulsion of antibiotics through multidrug resistance efflux pump systems belonging to the resistance-nodulation-division family (154). Therefore, these may be important mechanisms facilitating bacterial adaptive responses to hypoxia, increasing virulence and persistence in the diseased airways. There are also suggestions that local tissue hypoxia in the diseased lung is advantageous to anaerobic pathogens such as *P. aeruginosa* over other pathogens (155). For example, biofilm formation itself contributes to local hypoxia of the diseased CF lung, which correlates with increased dependency on systems that mediate the uptake of reduced ferrous iron (Fe^2+^) by *P. aeruginosa* (156). Future studies investigating the role of hypoxia in biofilm formation with the use of *ex vivo* models would be valuable to understand the mechanisms of biofilm formation in the hypoxic airways (157). Such models could also be used to investigate potential future biofilm-targeting therapeutics.

### Dysregulated Proteolytic Activity

During inflammatory processes, a plethora of toxic inflammatory by-products are released by immune cells. For example, neutrophils infiltrating the site of infection have been implicated in causing excessive tissue damage through release of proteases and reactive oxygen species (ROS) (158). Furthermore, macrophages produce matrix metalloproteinases (MMPs) during infectious processes, which can lead to excessive tissue damage (159). Therefore, an adequate protease-antiprotease balance is required to prevent excessive tissue damage and inflammation. Secretory leukocyte protease inhibitor (SLPI) is an important antiprotease, which prevents excessive tissue damage and inflammation (160). This protein also possesses key antimicrobial functions against Gram-negative and Gram-positive bacteria (161, 162). *In vivo* experiments have demonstrated that SLPI has the ability to dampen the macrophage associated inflammatory burden induced by LPS (163). Furthermore, the pathogenesis of chronic inflammatory respiratory diseases are thought to involve a protease-antiprotease imbalance (164, 165). Sputum samples from COPD patients display lower levels of SLPI during infectious exacerbations (166, 167), and lower levels of SLPI are associated with pronounced airway inflammation, susceptibility to infection, and disease severity (168, 169). It has been demonstrated that hypoxia downregulates the expression of SLPI in airway epithelial cells *via* the upregulation of transforming growth factor (TGF)-β (170). The expression and function of TGF-β is mediated by HIF-1α during hypoxia to promote cell growth and proliferation (171, 172). Furthermore, TGF-β has been implicated in the vascular remodeling of hypoxia-induced pulmonary hypertension and is also upregulated in the lungs of CF and COPD patients (173–176). Inhibition of SLPI by TGF-β *via* the SMAD signaling pathway has been demonstrated at both the RNA and protein level (177, 178). This modulation of complementary mechanisms by tissue hypoxia through alterations in TGF-β and thus SLPI expression could accentuate the protease-antiprotease imbalance and exacerbate inflammatory responses leading to increased tissue damage and progression of inflammatory lung diseases.

### Disrupted Airway Glucose Homeostasis

In the airways, the composition of the airway surface liquid (ASL) plays a critical role in the first line of defense against infection. In health, glucose concentrations in the fluid lining the ASL are maintained at 0.4 mM, about 12 times lower than the concentration of glucose in the bloodstream (179). This is an important airway defense mechanism against infection, limiting bacterial growth by restricting nutrient availability (180). However, in chronic inflammatory respiratory diseases including CF and COPD, the concentration of glucose in the ASL is increased (179, 181, 182). Disruption of airway glucose homeostasis increases the availability of glucose as a nutrient source in the ASL for bacterial pathogens. Consequently, this has the potential to support proliferation of bacteria able to utilize glucose as a carbon source, increasing bacterial loads and altering bacterial communities. Evidence from *in vitro* (181, 183–185), animal (186), and human studies (187), indicates that elevated ASL glucose stimulates the proliferation of *P. aeruginosa, S. aureus*, and other Gram-negative bacteria, which promote bacterial lung infections (188–190). Additionally, elevated levels of glucose in the ASL is associated with exacerbation, inflammatory markers and bacterial load in COPD (181). The principle mechanism thought to be limiting ASL glucose concentration are the epithelial TJs, which restrict paracellular glucose movement (191). It has previously been shown that airway epithelial cell cultures infected with *P. aeruginosa*, resulted in TJ protein disruption, which was associated with increases in paracellular glucose flux, indicating the importance of epithelial barrier integrity in glucose airway homeostasis (192). It is not yet known if hypoxia impacts ASL glucose concentrations but the presence of hypoxia in chronic inflammatory respiratory diseases and the profound impact of hypoxia on the epithelial barrier and TJ expression may indicate a role for tissue hypoxia and HIF-1. Increased glucose concentrations in the ASL may consequently facilitate nutrient availability for invading pathogens and increase their persistence in the airways. Thus, future experiments assessing the role of hypoxia and glucose airway homeostasis could be explored further in human *ex vivo* cell cultures.

### Enhanced Viral Replication in Epithelial Cells

Switching to glycolytic pathways may also be an important mechanism for viral replication in the lungs. During hypoxic stress, the rate of oxidative phosphorylation is reduced, switching cellular metabolism to the use of anaerobic glycolytic pathways *via* HIF-1α transcriptional activity (193). Airway epithelial cells respond in a similar fashion, displaying both aerobic and anaerobic glycolytic capacities (194). Ouiddir et al., demonstrated that airway epithelial cells exposed to hypoxia induced a three-fold increase in the expression of the glucose transporter GLUT1, at both the mRNA and protein level (195). The authors concluded that the epithelial cells ability to sustain ATP production during hypoxia was due to an increase in anaerobic glycolysis and increased glucose transport at the membrane level (195). Additionally, hypoxia has been shown to induce the expression and activation of key glycolytic enzymes important for the breakdown of glucose, including pyruvate kinase, lactate dehydrogenase (194), and glyceraldehyde phosphate dehydrogenase (GAPDH) (196). More recently it was discovered that airway epithelial cells exposed to hypoxia resulted in an increase in HIF-1α (197). In these hypoxic cells mitochondrial respiration was subsequently reduced as well as the rate of protein synthesis and demands for ATP, and the activity of GAPDH was increased (197). The influenza H1N1 virus has been found to exploit this mechanism, mimicking the hypoxic response to stabilize HIF-1α during infection of airway epithelial cells (198). This in turn, upregulates the expression of GLUT1, which may reprogram the host cellular glucose metabolism towards enhanced glycolysis to support nucleotide biosynthesis and lipogenesis for efficient viral replication. Rhinovirus infection has also been shown to induce metabolic alterations in host cells by increasing GLUT1 expression with increased glucose uptake and enhanced viral replication (199). More recently, it was demonstrated that monocytes infected with SARS-CoV-2 resulted in mitochondrial ROS-mediated stabilization of HIF-1α and increased glycolysis (200). The increase in glycolysis consequently promoted SARS-CoV-2 replication and cytokine expression. It is not yet known if similar processes take place in airway epithelial cells. However, it can be postulated that hypoxia in the airway during SARS-CoV-2 infection may increase the use of glycolytic enzymes through HIF-1 pathways which may support SARS-CoV-2 replication. In summary, tissue hypoxia and HIF-1 may play an important role in the pathogenesis of viral infections through the switching of glycolytic pathways which can support viral replication. This could be of particular importance in COVID-19 and respiratory diseases such as asthma and COPD where viral infections play a key role in exacerbations and disease progression (201, 202).

## Conclusions and Future Directions

Our understanding of the role of tissue hypoxia in mediating the epithelial-pathogen interactions in respiratory diseases is increasing. Hypoxia modulates several innate immune responses including impairing epithelial barrier function, reducing mucociliary clearance, modulating nutrient availability, and reducing protease inhibitors. Hypoxia could also play an important role in pathogen colonization of the lung epithelium through mediating bacterial adherence, internalization, biofilm formation, and viral replication. This review has highlighted potential future experimental work that may aid identification of new drug targets and the development of novel therapeutics. Developing a deeper understanding of the oxygen microenvironment within the lung is now key to create appropriate models and more accurately delineate mucosal host-pathogen interactions. Furthermore, understanding the impact of hypoxia-mediated modulation of transcription factors and the potential numerous implications for respiratory disease is essential. A greater understanding of the balance between beneficial and detrimental HIF-1 activation is also needed. Indeed, HIF-1 activity is critical for host response to pathogens and helps shape the innate and adaptive response. Thus, not all HIF-1 activation can be considered harmful and the therapeutic inhibition of this pathway must be balanced against its beneficial contribution. Nonetheless, targeting the HIF signaling pathway in chronic respiratory disease may still hold promise in effectively managing or delaying the progression of disease (203). Novel therapeutics could be developed which specifically interfere with mRNA expression, protein synthesis, protein degradation, protein dimerization, DNA binding or transcriptional activity of HIF-1. Several *in vitro* studies have used various methods to inhibit or silence HIF-1 and have ameliorated its deleterious effects in disease models (73, 114, 140, 204). Furthermore, the use of HIF-1α inhibitors for the treatment of patients infected with COVID-19 has recently been highlighted (205). These potential strategies provide interesting opportunities to potentially modulate tissue hypoxia and lung mucosal host-pathogen interactions.

## Author Contributions

LP: conceptualization, investigation, literature searching, analysis, project administration, writing original draft, reviewing, and editing. KS: supervision, conceptualization, reviewing, and editing. MS: supervision, conceptualization, reviewing, and editing. AW: supervision, reviewing, and editing. TW: supervision, conceptualization, reviewing, and editing. All authors contributed to the article and approved the submitted version.

## Funding

LP is funded by a BBSRC-GSK iCASE studentship.

## Conflict of Interest

TW reports grants and personal fees from AstraZeneca, personal fees and other from MMH, grants and personal fees from GSK, personal fees from BI and grants and personal fees from Synairgen, outside the submitted work. KS reports grants from AstraZeneca outside the submitted work.

The remaining authors declare that the research was conducted in the absence of any commercial or financial relationships that could be construed as a potential conflict of interest.
